# Whole-body and adipose tissue-specific mechanisms underlying the metabolic effects of fibroblast growth factor 21 in the Siberian hamster

**DOI:** 10.1016/j.molmet.2019.10.009

**Published:** 2019-11-09

**Authors:** Jo E. Lewis, Chloe Monnier, Hayley Marshall, Maxine Fowler, Rebecca Green, Scott Cooper, Aristeidis Chiotellis, Jeni Luckett, Alan C. Perkins, Tamer Coskun, Andrew C. Adams, Ricardo J. Samms, Francis J.P. Ebling, Kostas Tsintzas

**Affiliations:** 1 Institute of Metabolic Sciences and MRC-Metabolic Diseases Unit, University of Cambridge, Cambridge, CB0 0QQ, UK; 2 School of Life Sciences, University of Nottingham Medical School, Queen's Medical Center, Nottingham, NG7 2UH, UK; 3 Radiological Sciences, School of Medicine, University of Nottingham, Queen's Medical Center, Nottingham, NG7 2UH, UK; 4 Eli Lilly and Company, Lilly Research Laboratories, Indianapolis, IN, 46285, USA

**Keywords:** FGF21, β-klotho, White adipose tissue, Brown adipose tissue, Glucose uptake, Lipid uptake

## Abstract

**Objective:**

Fibroblast growth factor 21 (FGF21) has been shown to rapidly lower body weight in the Siberian hamster, a preclinical model of adiposity. This induced negative energy balance mediated by FGF21 is associated with both lowered caloric intake and increased energy expenditure. Previous research demonstrated that adipose tissue (AT) is one of the primary sites of FGF21 action and may be responsible for its ability to increase the whole-body metabolic rate. The present study sought to determine the relative importance of white (subcutaneous AT [sWAT] and visceral AT [vWAT]), and brown (interscapular brown AT [iBAT]) in governing FGF21-mediated metabolic improvements using the tissue-specific uptake of glucose and lipids as a proxy for metabolic activity.

**Methods:**

We used positron emission tomography-computed tomography (PET-CT) imaging in combination with both glucose (^18^F-fluorodeoxyglucose) and lipid (^18^F-4-thiapalmitate) tracers to assess the effect of FGF21 on the tissue-specific uptake of these metabolites and compared responses to a control group pair-fed to match the food intake of the FGF21-treated group. In vivo imaging was combined with ex vivo tissue-specific functional, biochemical, and molecular analyses of the nutrient uptake and signaling pathways.

**Results:**

Consistent with previous findings, FGF21 reduced body weight via reduced caloric intake and increased energy expenditure in the Siberian hamster. PET-CT studies demonstrated that FGF21 increased the uptake of glucose in BAT and WAT independently of reduced food intake and body weight as demonstrated by imaging of the pair-fed group. Furthermore, FGF21 increased glucose uptake in the primary adipocytes, confirming that these in vivo effects may be due to a direct action of FGF21 at the level of the adipocytes. Mechanistically, the effects of FGF21 are associated with activation of the ERK signaling pathway and upregulation of GLUT4 protein content in all fat depots. In response to treatment with FGF21, we observed an increase in the markers of lipolysis and lipogenesis in both the subcutaneous and visceral WAT depots. In contrast, FGF21 was only able to directly increase the uptake of lipid into BAT.

**Conclusions:**

These data identify brown and white fat depots as primary peripheral sites of action of FGF21 in promoting glucose uptake and also indicate that FGF21 selectively stimulates lipid uptake in brown fat, which may fuel thermogenesis.

## Highlights

• FGF21 increases glucose and lipid uptake in adipose tissue.
• The selective FGF21-induced increase in lipid uptake in BAT may fuel thermogenesis.
• Unlike BAT, glucose uptake in WAT may be used for lipogenesis.

## 1 Introduction

Fibroblast growth factor 21 (FGF21) is a member of the FGF superfamily that regulates metabolic homeostasis and is a potential therapeutic target for the treatment of metabolic syndrome [1]. Pharmacological administration of FGF21 to obese and diabetic animal models results in multiple metabolic benefits including improved glucose homeostasis via increased insulin sensitivity and reduced body weight [[2], [3], [4]]. FGF21's pleiotropic effects are determined by the expression of FGFR1 and an obligate co-receptor, β-klotho (KLB) [5]. However, FGF21 can also activate FGFR3c and KLB [6]. The restricted expression of KLB in adipose tissue (AT) and certain regions of the brain dictate the tissue-specific actions of FGF21. Multiple studies have implicated the importance of AT and the central nervous system (CNS) for FGF21 biology. Deletion of the receptor isotype FGFR1 or KLB from AT impairs the insulin-sensitizing effects of FGF21, and the effects of antibodies targeting FGFR1 are impaired in lipodystrophic mice [[7], [8], [9], [10]]. It was recently shown in mice genetically engineered to delete KLB signaling from white AT (WAT) that AT is required for the acute insulin-sensitizing effects of FGF21 [11]. Previously continuous intracerebroventricular (ICV) infusion of FGF21 was shown to increase food intake and energy expenditure in diet-induced obese rats, while mice lacking KLB in the suprachiasmatic nucleus (SCN) and hindbrain were refractory to the effects of FGF21 [12,13]. Furthermore, infusion of FGF21 into the lateral ventricle in mice was shown to stimulate browning of AT, which raises the possibility that some of the effects of FGF21 on AT are centrally mediated [14].

We previously studied the biology of FGF21 action in the Siberian hamster, a natural model of adiposity that increases body weight as it accumulates AT in long day summer photoperiods (LD) but catabolizes these depots to support winter survival in short photoperiods (SD) [15]. Hamsters in the LD state offer the possibility of studying the actions of FGF21 in fat animals without the confounding effects of the development of insulin resistance. We previously showed that FGF21 treatment suppresses appetite and promotes lipid oxidation in the LD state, but these effects are lost in the winter SD state, which is associated with reduced adiposity [16]. Although the mechanisms underlying the reduced responsiveness to FGF21 in the SD state are not yet known, we also demonstrated that the effects of FGF21 are attenuated in aged Siberian hamsters, which also display reduced adiposity [17], reinforcing the importance of AT in mediating the metabolic effects of FGF21. In humans with type 2 diabetes (T2D), chronic administration of FGF21 analogs lowers body weight and improves plasma lipid profiles [18,19]. However, which specific adipose tissue depots may account for the metabolic effects of FGF21 and the underlying mechanisms via which these are achieved have yet to be fully elucidated [20].

In the present study, we sought to determine the relative importance of different depots of AT (interscapular brown [iBAT], interscapular subcutaneous white [sWAT], and peri-renal [visceral] white AT [vWAT]) in governing FGF21-mediated metabolic improvements using a combination of in vivo studies utilizing quantitative PET-CT imaging with both glucose and lipid tracers to assess the location and magnitude of their uptake with ex vivo, metabolic, and molecular biology approaches. By combining whole-body physiology along with mechanistic studies, we demonstrate the adipose tissue-specific capacity for glucose and lipid uptake and metabolism and the importance of adiposity, and in particular KLB expression, in governing FGF21 responsiveness.

## 2 Methods

### 2.1 Animals

Adult male Siberian hamsters were obtained from a colony maintained at the University of Nottingham Biomedical Services Unit. All of the studies were approved by the University of Nottingham Local Ethical Review Committee and conducted in accordance with the UK Animals (Scientific Procedures) Act of 1986 (project license: PPL 40/3604 and PFBB5B31F). The Siberian hamsters were housed as previously described [21]. Pair-fed (PF) hamsters were food restricted (twice daily) to the average intake of FGF21-treated animals (offset by 48 h) as previously described [22].

### 2.2 Chronic FGF21 (LY2405319) treatment

Prior to surgery, the animals were singly housed to accurately determine food intake. Age-matched animals in the LD (16 h light, 8 h dark) and SD (16 h dark, 8 h light, lights off at 11:00) condition received a subcutaneously implanted ALZET osmotic mini-pump (Model 1007D, Charles River) releasing vehicle (saline) or FGF21 (LY2405319; 3 mg/kg/day) for 7 days as previously described [17]. This infusion paradigm was expected to increase the circulating FGF21 concentrations by at least 2.5-fold over the course of the study. Food intake and body weight were measured daily. Three days post-surgery, the animals were transferred to metabolic cages (Columbus, Linton Instrumentation, UK).

### 2.3 Metabolic cages

Oxygen consumption and carbon dioxide production (used to calculate the respiratory exchange ratio [RER] and energy expenditure [EE]) were measured concurrently in a modified open-circuit calorimeter known as the comprehensive laboratory animal monitoring system (CLAMS) as previously described [21].

### 2.4 Assessment of body composition and whole-body glucose and lipid uptake

Six days post-surgery, morphologic and molecular imaging was undertaken using a nanopositron emission tomography-computed tomography (nanoPET-CT) imaging scanner (Mediso Medical Imaging Systems, Budapest, Hungary) to quantify the body composition, and tissue-specific glucose and lipid uptake using ^18^F-fluorodeoxyglucose (^18^F-FDG) (10 MBq per animal) and ^18^F-4-thiapalmitate (^18^F-PAL) (8 MBq per animal), respectively, as previously described [23,24]. The magnitude of tissue-specific ^18^F-FDG and ^18^F-PAL uptake was expressed as the standard uptake value (SUV) defined as the average ^18^F activity in each region of interest (in kBq/cm^3^) divided by the injected dose (kBq) and multiplied by the body weight of each animal (kg).

### 2.5 Primary adipocytes

All reagents were obtained from Sigma unless otherwise stated. Eight-to 12-week-old male LD Siberian hamsters were used to obtain primary adipocytes. Briefly, the fat pads (iBAT, sWAT, and vWAT) were removed, chopped, and digested using collagenase D and dispase II at 37 °C with constant agitation for 1 h in PBS containing 10 mM CaCl_2_. The digestion was stopped by adding DMEM/F12, centrifuging at 900×*g* for 10 min, and resuspension of the pellets in RBS Lysis Buffer Solution, centrifuging at 900×*g* for 10 min, and resuspension in DMEM/F12 containing 10% FBS and pen/strep. The media were changed every 2 days until 95% confluent. To differentiate the BAT cultures, the cells were treated with 5 μg/ml insulin, 1 nM T3, 125 μM indomethacin, 2 μg/ml dexamethasone, 500 μM IBMX, and 0.5 μM Rosi for 48 h. The WAT cultures were treated with 5 μg/ml insulin, 2 μg/ml dexamethasone, and 500 μM IBMX only for 48 h. On day 2, the BAT cultures were treated with 5 μg/ml insulin, 1 nM T3, and 0.5 μM Rosi, while the WAT cultures were treated with 2 μg/ml dexamethasone only. On days 4, 6, 8, and 10, the cells were maintained in DMEM/F12, 10% FBS, and pen/step before treatment with 100 nM LY2405319 for 48 h. Non-insulin stimulated glucose uptake (normalized for protein content) was determined using [^3^H]2-deoxyglucose as previously described [25].

### 2.6 Molecular and biochemical analysis

The animals were subsequently euthanized via an intraperitoneal injection of sodium pentobarbital (Euthatal, Rhone Merireux, Harlow, UK). Samples of the brain, liver, iBAT, sWAT, and vWAT were collected and snap frozen on dry ice and stored at −80 °C until required. Total RNA, protein (see Supplementary Table 1 for antibody information and Supplementary Fig. 4 for examples of Western blotting), and TAG were extracted and analyzed as previously described [23,26]. Glycerol-3-phosphate was determined according to the manufacturer's instructions (Sigma MAK207, UK). Pyruvate dehydrogenase complex (PDC) activity was determined as previously described [27].

### 2.7 Statistical analysis

Descriptive statistics (means ± SEM) were generated using GraphPad Prism (Prism 7.0, GraphPad, San Diego, CA, USA). Data were analyzed using two-way ANOVA, one-way ANOVA, or Student's t-test as appropriate. No animals were excluded from the analyses. Statistical significance was considered at p < 0.05.

## 3 Results

### 3.1 Effects of FGF21 on body weight, activity, substrate oxidation rates, and tissue thermogenic markers (Figure 1)

**Figure 1:**
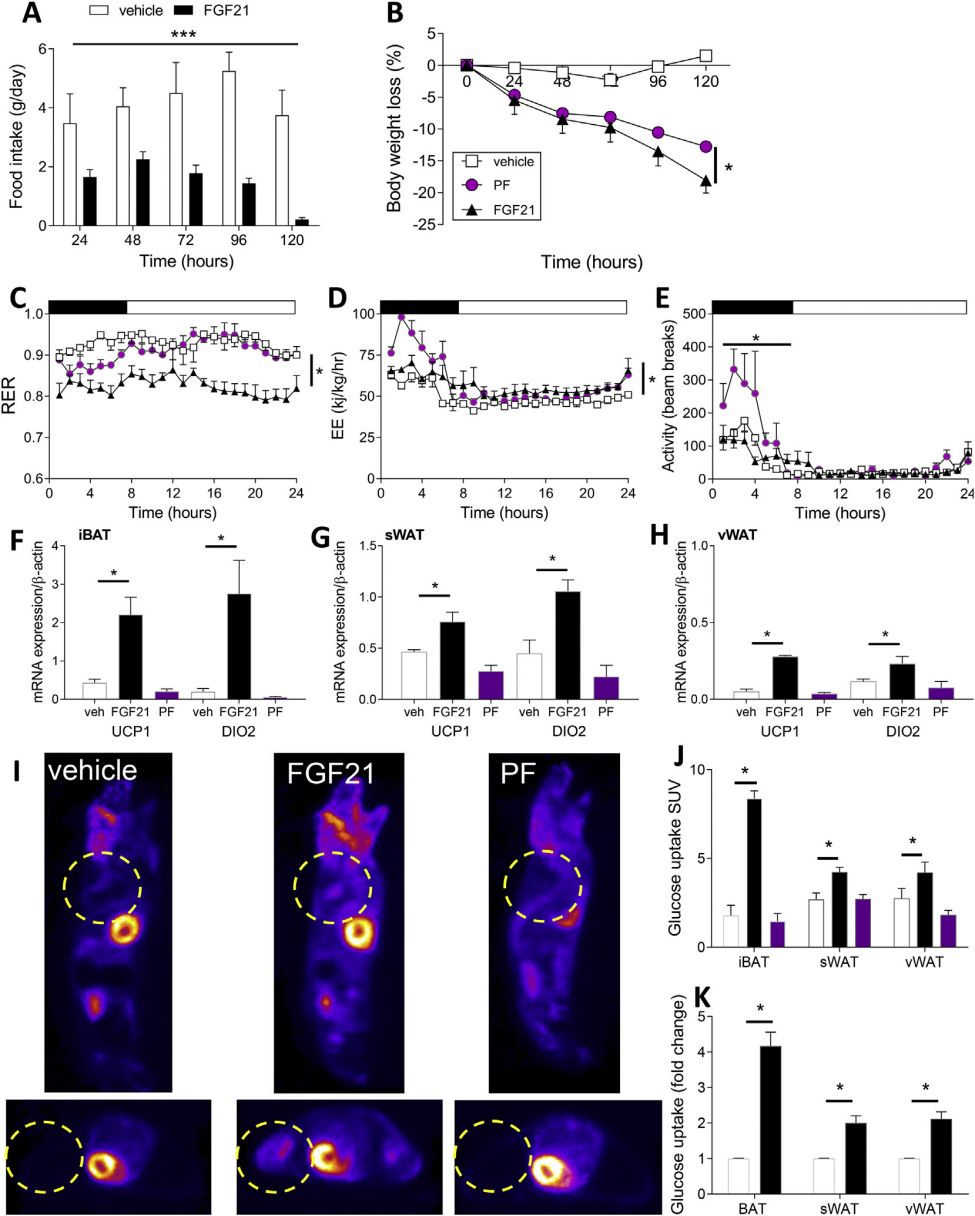
**FGF21 reduces body weight via reduced food intake and increased energy expenditure in Siberian hamsters in long days (LD).** (A) Food intake, (B) body weight, (C) RER, (D) energy expenditure, (E) ambulatory activity, (F, G, and H) UCP1 and DIO2 mRNA expression in AT, and (I and J) glucose uptake of Siberian hamsters treated with FGF21, vehicle, or pair-fed to match the food intake of the FGF21-treated group. Data are mean ± SEM. N = 4/group. *p < 0.05, ***p < 0.001. (K) Glucose uptake of primary adipocytes derived from interscapular brown adipose (iBAT), interscapular subcutaneous white adipose (sWAT), or visceral (perirenal) white adipose (vWAT) treated with FGF21. N = 8 per treatment. Data presented as mean ± SEM. *p < 0.05 vs vehicle control. (For interpretation of the references to color in this figure legend, the reader is referred to the Web version of this article.)

Chronic treatment with FGF21 significantly reduced the body weight and fat mass in the Siberian hamsters maintained in the LD fat state (Figure 1B). Body weight loss was associated with a lowered daily caloric intake and increased energy expenditure (Figure 1A,D). The hamsters maintained in LD but pair-fed (PF) to match the intake of the hamsters undergoing FGF21 treatment also lost body weight; however, the magnitude of weight loss was attenuated when compared to the ab libitum fed FGF21-treated animals (PF = 12.8 vs FGF21 = 18.1%, p < 0.05, Figure 1B). Treatment with FGF21 reduced RER in the hamsters maintained in LD, indicating an increase in whole-body lipid oxidation (Figure 1C). This was a consequence of the FGF21 treatment per se, as the PF animals maintained higher RER values that were similar to the ab libitum fed vehicle control group (Figure 1C). Interestingly, the PF animals also demonstrated increased EE; however, this was limited to the dark phase (Figure 1D) and appeared to be a consequence of increased ambulatory activity (Figure 1E). The ambulatory activity of the FGF21-treated animals and vehicle-treated controls was comparable (Figure 1E). The whole-body increase in energy expenditure was associated with a significant increase in UCP1 and DIO2 mRNA expression in iBAT, sWAT, and vWAT in the FGF21-treated animals (Figure 1F–H). In the PF group, these thermogenic markers were significantly reduced compared to the vehicle-treated control animals (Figure 1F–H).

### 3.2 Effects of FGF21 on tissue glucose uptake (Figure 1 and Supplemental Fig. 1A)

We assessed tissue- and depot-specific glucose uptake in response to treatment with FGF21 in vivo using nanoPET-CT and ^18^F-FDG. Treatment with FGF21 increased the glucose uptake in iBAT, sWAT, and vWAT compared to the vehicle-treated controls (Figure 1I,J). The increase in the sWAT and vWAT depots was similar. This was a direct effect of treatment, rather than a consequence of reduced food intake, as glucose uptake in the PF group was similar to the ab libitum fed control group (Figure 1I,J). Glucose uptake in response to treatment was unaffected in the other tissues, including the brain, liver, muscle, and testes (Supplemental Fig. 1A). To investigate whether the AT response was a direct effect of the FGF21 treatment at the level of the adipocyte, we treated the hamster primary adipocytes differentiated from the stromal avascular cells isolated from iBAT, sWAT, and vWAT with a pharmacological dose of FGF21 (100 nM for 48 h). FGF21 increased the glucose uptake significantly in the cultures from both the tissues and depots (Figure 1K). In line with the whole-tissue findings, treatment with FGF21 also induced a 10- and 3-fold increase in UCP1 mRNA in the iBAT and WAT cultures, respectively (p < 0.01).

### 3.3 Effects of FGF21 on tissue-signaling proteins, transporters, and enzymes (Figure 2, Supplemental Fig. 3)

**Figure 2:**
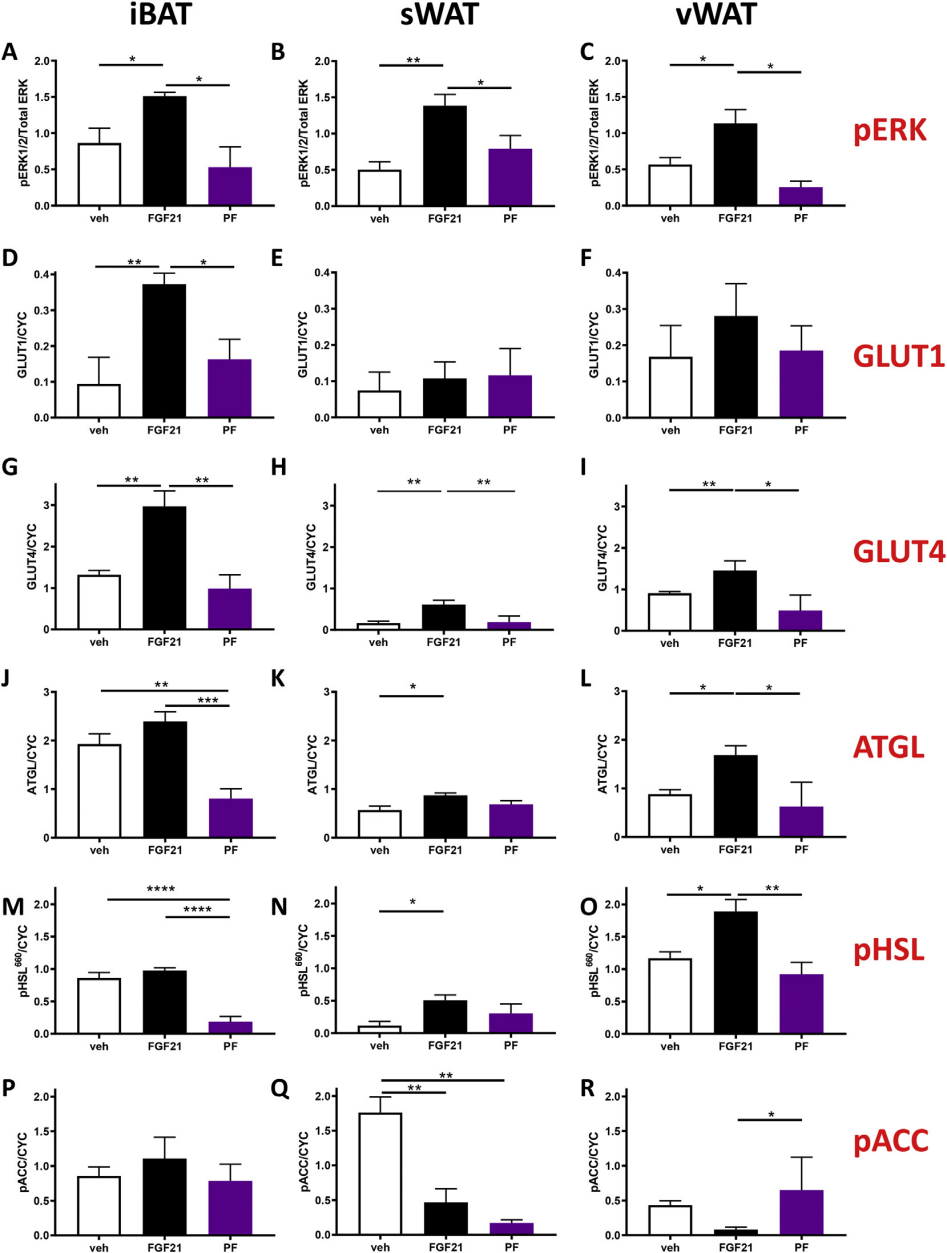
**FGF21-stimulated glucose uptake in adipose tissue relates to GLUT1 and GLUT4 protein expression in vivo.** (A–C) pERK1/2 activation, (D–F) GLUT1 expression, (G–I) GLUT4 expression, (J–L) ATGL expression, (M–O) pHSL660, and (P–R) pACC in iBAT, sWAT, and vWAT in response to treatment with FGF21 in vivo. Data are mean ± SEM. N = 4/group. Statistical analyses conducted using one-way ANOVA with Tukey's multiple comparison test. *p < 0.05, **p < 0.01, ***p < 0.001, ****p < 0.0001.

Tissue-specific phosphorylation events downstream of the FGFR1c-KLB receptor complex, namely pERK1/2, were significantly increased in the iBAT, sWAT, and vWAT of the FGF21-treated animals (Figure 2A,B, and C). Treatment with FGF21 also increased the GLUT1 protein content (a downstream target of ERK1/2) in iBAT (Figure 2D). This increase was not apparent in either WAT depot (Figure 2E,F). However, GLUT4 protein (also a target of ERK1/2) was significantly increased in iBAT and both WAT depots (Figure 2G–I). There were no changes in either GLUT1 or GLUT4 protein abundance in response to PF in either tissue. ERK phosphorylation also contributes to AT lipolysis [28,29]. In the present study, key markers of lipolytic capacity, namely ATGL and pHSL^660^, were unaffected by treatment in iBAT (Figure 2J,M); however, the PF group showed a significant reduction in both markers. In contrast, treatment with FGF21 increased the ATGL content and pHSL^660^ in sWAT and vWAT (Figure 2K,L, 2N, and O, respectively). These responses do not appear to be a consequence of reduced food intake as there were no significant changes in those lipolytic proteins in the PF group compared to the control (saline) group. The phosphorylation of ACC (pACC), a key enzyme in tissue lipogenesis that controls the conversion of acetyl-CoA to malonyl-CoA, was unaffected in iBAT by FGF21 treatment or reduced food intake in the PF group (Figure 2P). However, pACC was reduced in WAT and therefore its activity increased in response to FGF21 treatment (Figure 2Q–R), with a more pronounced effect observed in sWAT compared to vWAT. We then assessed the activation of the PDC, which controls the conversion of pyruvate to acetyl-CoA that is the substrate for ACC. Both the FGF21-treated and PF animals demonstrated increases (0.49 ± 0.10 and 0.33 ± 0.07 nmol acetyl CoA/mg/min, p < 0.05, respectively) in PDC activity in vWAT compared to the control (saline) group (0.07 ± 0.01 nmol acetyl CoA/mg/min). In BAT, there was a tendency (p = 0.09) for an increase in PDC activity in the FGF21 (1.48 ± 0.45 nmol acetyl CoA/mg/min) but not PF group (0.70 ± 0.32 nmol acetyl CoA/mg/min) when compared with saline (0.56 ± 0.10 nmol acetyl CoA/mg/min).

### 3.4 Effects of FGF21 on tissue metabolites (Figure 3)

**Figure 3:**
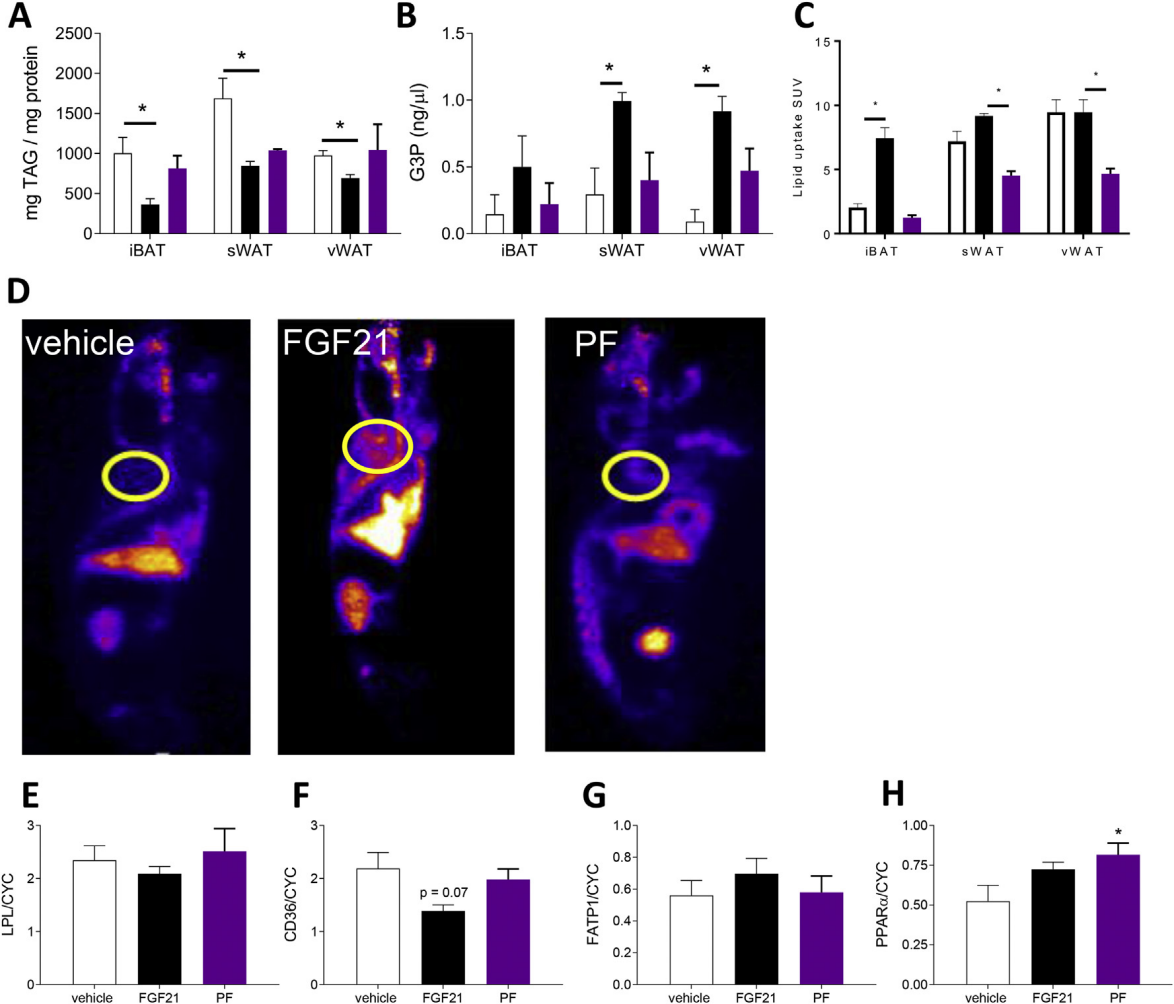
**FGF21-stimulated lipid uptake in iBAT.** (A) TAG and (B) G3P content of iBAT, sWAT, and vWAT and (C) lipid uptake in iBAT, sWAT, and vWAT in response to treatment with FGF21 or vehicle or in pair-fed controls (PF). Data are mean ± SEM. N = 4/group. Statistical analyses conducted using one-way ANOVA with Tukey's multiple comparison test. *p < 0.05. (E) LPL, (F) CD36, (G) FATP1, and (H) PPARα expression in iBAT in response to treatment with FGF21 (vs saline controls). N = 4/group. Data are mean ± SEM. N = 4/group. Statistical analyses conducted using one-way ANOVA with Tukey's multiple comparison test. *p < 0.05.

Treatment with FGF21 reduced the TAG content in iBAT and both WAT depots (Figure 3A). Cold-activated BAT uses large amounts of TAG for thermogenic purposes, and thus there is a biological need for efficient mechanism(s) ensuring the continuous replenishment of intracellular lipid stores. This includes glucose uptake that is used for de novo lipogenesis and glycerol synthesis [30]. This study measured the levels of glycerol-3-phosphate (G3P), a key intermediate in lipogenesis that is required for the re-esterification of TAG. In line with increased glucose uptake and ACC activity, treatment with FGF21 resulted in a significant increase in the G3P concentration in sWAT and vWAT, but not iBAT (Figure 3B). In the PF control group, the levels of G3P were comparable to the saline-treated animals.

### 3.5 Effects of FGF21 on tissue lipid uptake and associated proteins (Figure 3 and Supplemental Fig. 1B)

Because both glucose and fatty acids are important substrates for the replenishment of intracellular lipid stores in adipose tissue, we sought to ascertain the impact of FGF21 on tissue-specific lipid uptake in vivo. Utilizing nanoPET-CT and infusion of ^18^F-PAL, we demonstrated that treatment with FGF21 significantly increased lipid uptake in BAT from the hamsters held in LD (Figure 3C). This was a direct effect of treatment, rather than a consequence of reduced food intake as demonstrated by the absence of lipid uptake in BAT in the PF group. There was no effect of FGF21 treatment on WAT; however, the PF group demonstrated reduced lipid uptake in that tissue. Furthermore, lipid uptake in response to FGF21 treatment was unaffected in the other tissues, including the brain, liver, quadriceps muscle, and testes (Supplemental Fig. 1B). The selective increase in lipid uptake in BAT in response to FGF21 treatment was not associated with altered mRNA expression of key proteins implicated in lipid transport into that tissue, namely LPL, CD36, and FATP1 (Figure 3E–G) previously implicated in lipid disposal in BAT and WAT [31]. However, there was an increase in the protein content of PPARα, a transcriptional regulator of FGF21, in BAT in the PF group compared with the control animals (Figure 3H).

### 3.6 Importance of adiposity and KLB expression in governing FGF21 responsiveness (Figure 4 and Supplemental Fig. 2)

Having described the distinct roles of different AT depots in mediating the thermogenic and metabolic effects of FGF21 in the Siberian hamsters maintained in the LD fat state, we next sought to determine the importance of the state of adiposity on these effects taking advantage of our preclinical natural model of adiposity. We showed transfer to SD for 12 weeks was associated with a reduction in body weight. In line with our previous studies, the effects of treatment with FGF21 were lost in this SD group at the nadir of their body weight cycle: food intake, body weight, RER, energy expenditure, UCP1 and DIO2 gene expression in AT, and tissue glucose uptake were all unaffected by FGF21 treatment (Supplemental Fig. 2).

**Figure 4:**
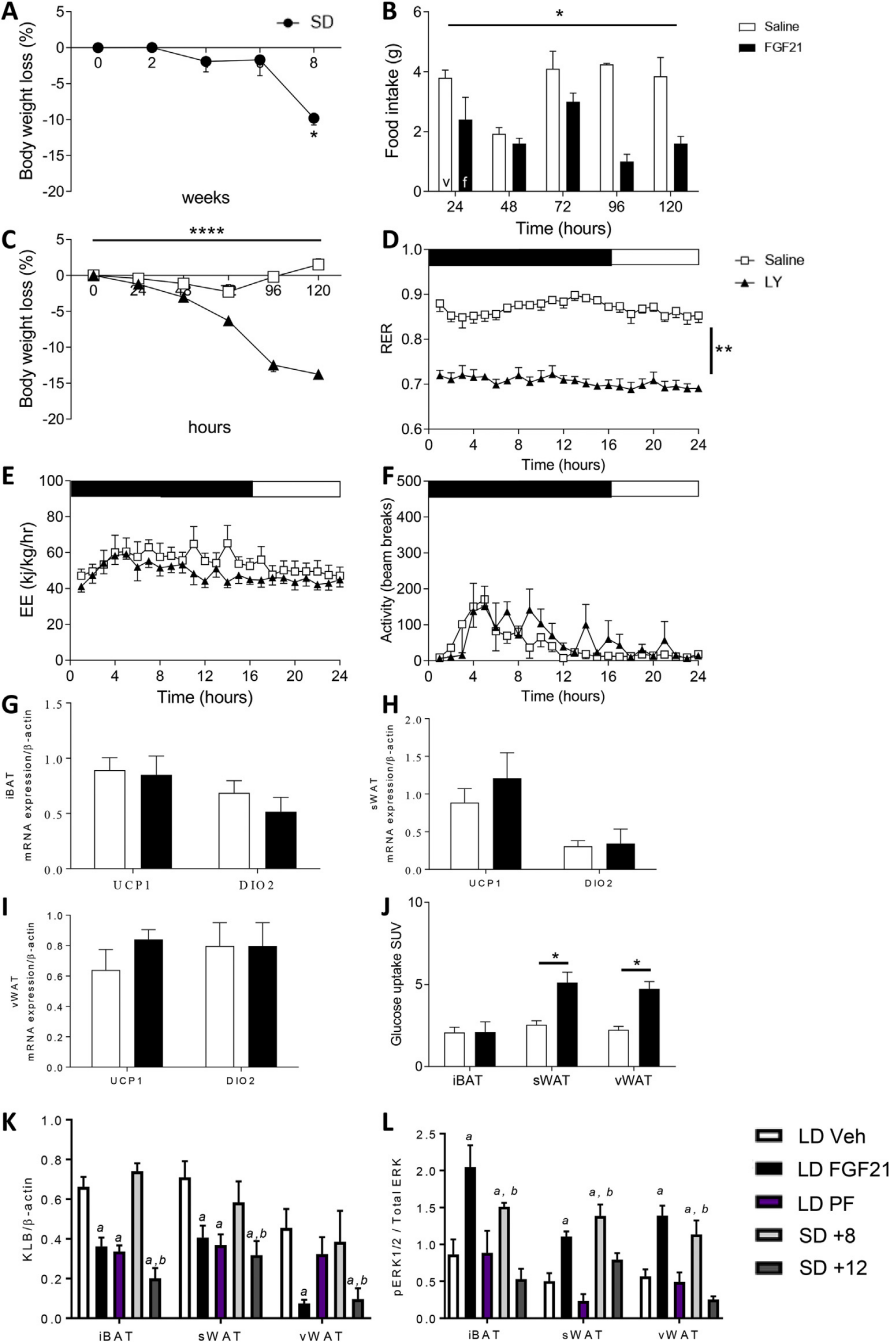
**FGF21 reduces body weight via reduced food intake in Siberian hamsters in short days (SD) before body weight reaches a seasonal nadir.** (A) Body weight loss in response to exposure to SD. (B) Food intake, (C) body weight, (D) RER, (E) energy expenditure, (F) ambulatory activity, (G–I) UCP1 and DIO2 mRNA expression in AT, and (J) glucose uptake in the Siberian hamsters treated with FGF21 or vehicle. Data are mean ± SEM. N = 4/group. Statistical analyses conducted using two-way ANOVA, one-way ANOVA, or Students' T-test as appropriate. *p < 0.05, ***p < 0.001, ****p < 0.0001. (K) KLB expression and (L) pERK1/2 in the Siberian hamsters in LD, treated with FGF21, PF to treatment group, or exposed to SD (8 and 12 weeks, respectively). ^a^p < 0.05 vs LD, ^b^p < 0.05 vs SD + 8 weeks or 12 weeks, respectively.

We subsequently explored the mechanism underlying this FGF21-resistant state in the SD animals and the importance of tissue glucose uptake in the increase in whole-body EE. We exposed a second group of Siberian hamsters to SD for 8 weeks. At this point, their body weight was significantly reduced (Figure 4A) compared to that during LD, but the animals were not at the nadir of the seasonal body weight cycle observed after 12 weeks of exposure to SD. At this stage of the seasonal cycle, treatment with FGF21 still significantly reduced food intake and body weight (Figure 4B,C). Furthermore, RER was significantly reduced (Figure 4D), indicating an increase in whole-body lipid oxidation. However, the effects of FGF21 on whole-body energy expenditure previously observed in LD were lost (Figure 4E). As with the hamsters studied in LD, there was no effect of FGF21 treatment on their ambulatory activity (Figure 4F). In addition, UCP1 and DIO2 expression in BAT and WAT was unaffected by treatment (Figure 4G–I). FGF21 treatment increased the glucose uptake in sWAT and vWAT but not iBAT in this cohort (Figure 4J). This suggests that the changes in glucose uptake in WAT were independent of the changes in EE in this tissue, and instead the FGF21-induced increase in EE in the LD animals was likely associated with the increased thermogenesis in BAT. In the absence of treatment with FGF21, the KLB levels in WAT in the hamsters exposed to SD for 8 weeks were comparable to the LD animals; while at the nadir of body weight (12 weeks post-exposure to SD), KLB was significantly reduced in iBAT, sWAT, and vWAT (Figure 4K). KLB was also reduced in the PF and FGF21 groups (Figure 4K); in the latter group, the weight loss was similar to the SD group (PF = −12.8%, FGF21 = −18.1%, SD + 8 = −9.8%, and SD + 12 week group = −21.0%). This reduced KLB in tissues in the SD + 12 week group was associated with a reduced pERK1/2 response to FGF21 (Figure 4L).

## 4 Discussion

This study provides novel insights into whole-body and adipose tissue-specific depot mechanisms governing the metabolic effects of FGF21 in the Siberian hamster, a seasonal model of adiposity. The selective increase in glucose uptake in the adipose tissue, which was a direct consequence of FGF21 treatment rather than a result of the associated changes in food intake, energy expenditure, and body weight, appeared to be differentially regulated in WAT and BAT. In addition, we demonstrated that lipid was exclusively taken up by BAT in response to treatment with FGF21, possibly to fuel thermogenesis either directly by being oxidized or indirectly by replenishing the TAG stores used for thermogenesis. This lipid was most likely supplied by WAT, as the markers of lipolysis in both sWAT and vWAT were upregulated in response to FGF21 treatment. However, we also demonstrated increased markers of TAG synthesis and de novo lipogenesis in both WAT depots, but not BAT, as a result of treatment with FGF21. In the absence of lipid uptake in WAT, our data suggest that the glucose uptake in this tissue may be used to supply both fatty acyl CoAs through the de novo lipogenesis pathway and the G3P required for the re-esterification of the glycerol backbone to TAG to provide further lipid stores for subsequent utilization. In hamsters in LD, increased EE following treatment with FGF21 was associated with an increased glucose uptake in BAT, but both effects were lost in SD. Finally, we demonstrated that at the nadir of the bodyweight cycle, there was a reduction in the expression of KLB associated with the loss of ERK1/2 phosphorylation in AT, altering its responsiveness to FGF21. Interestingly, KLB was significantly reduced in the PF group, suggesting that weight loss per se altered the expression of this obligate co-receptor for FGF21.

FGF21 was previously shown to be a potent regulator of glucose uptake in 3T3-L1 adipocytes via GLUT1 [32]. Furthermore, FGF21 acts as an insulin sensitizer in mice to overcome peripheral insulin resistance induced by fasting in mice, an effect that is decreased in BAT of liver-specific FGF21 KO mice [4]. The findings from the present study extend those observations by combining mechanistic in vitro studies with an in vivo model of seasonal adiposity using PET-CT measurements to simultaneously assess in real time both the glucose and lipid uptake responses to FGF21 treatment in multiple tissues and different depots of AT. In the LD state, treatment with FGF21 significantly increased the glucose uptake in BAT and both depots of WAT, which was a direct effect of treatment as demonstrated by the responses of the PF group and data from the primary adipocyte cultures. The mechanism by which glucose was taken up and its fate, however, were differentially regulated. In BAT, we observed increases in GLUT1 and GLUT4 protein content, suggesting insulin-independent and insulin-dependent mechanisms, respectively. FGF21-induced GLUT1 expression in the adipocytes was mediated by the Elk-1/SRF signaling cascade and was blunted in the diet-induced obese mice [33]. In sWAT and vWAT, the increase in glucose uptake was mediated by GLUT4 only. Overexpression of FGF21 has been previously shown to increase GLUT4 mRNA expression in adipose tissue [34]. In the present study, we extended that observation by showing an increase in the protein content of GLUT4 in both the subcutaneous and visceral WAT depots as well as BAT [35]. It should be noted that BAT depots in rodents and humans are highly insulin sensitive. Indeed, insulin administration enhances glucose uptake in BAT in response to FGF21 treatment [36]. In the present study, the magnitude of the effect of FG21 on the glucose uptake in BAT was 2-fold higher than WAT, which may be explained by the observation that GLUT4 is expressed at lower levels in WAT and our data in the Siberian hamster supports this [37]. Furthermore, this may explain why humans, who possess relatively less BAT, demonstrate less profound glucose lowering in response to FGF21 treatment compared to rodents [18,19]. However, human BAT has been shown to efficiently utilize fatty acids in vivo, and glucose uptake in BAT is not impaired in overweight subjects with T2D [38,39]. Consistent with the FGF21-stimulated lipid uptake in BAT in the present study, the blood lipid profile is improved in humans treated with FGF21 analogs [18,19]. Further clinical work is required to determine the contribution of BAT in pre-clinical models and human subjects (reviewed in [40]).

The increase in glucose uptake in WAT appears to contribute to the regeneration of TAG. Indeed, treatment with FGF21 was shown to activate ERK phosphorylation in that tissue and was associated with increased content/activity of key markers of lipolysis (ATGL and pHSL), de novo lipogenesis (ACC), and glycerol synthesis (G3P). The increase in PDC activity in WAT, which controls the conversion of pyruvate to acetyl-CoA, the substrate for ACC, further supports this notion. In contrast, the absence of effects of FGF21 on pACC and G3P in BAT, along with the tendency for an increase in PDC activity, suggests that most glucose taken up is oxidized in that tissue and hence, unlike WAT, de novo lipogenesis and glycerol synthesis do not substantially contribute to replenishment of intracellular lipid stores under those conditions.

We also demonstrated that treatment with FGF21 increased lipid uptake in BAT alone; we suggest the primary purpose of the lipid taken up is to both replenish the intracellular TAG stores in this tissue and fuel the increase in EE through thermogenesis. FGF21 may promote fatty acid uptake in adipocytes directly or indirectly via insulin sensitization; uptake was previously shown to be mediated via fatty acid transporters such as CD36 or FATP1 in adipocytes [31,41]. Although the findings from the present study indicate that the FGF21-induced uptake of lipid in BAT was not mediated by upregulation in the protein content of CD36, FATP1, or LPL, the possibility that changes in their intrinsic activity may play a role cannot be ruled out. The selective utilization of plasma-derived lipids in BAT, without further significant storage in WAT, may contribute not only to lipid lowering effects previously reported but also to the weight loss and insulin sensitization effects of FGF21 [31]. In addition, the increase in EE following treatment with FGF21 in the LD animals was associated with an increase in glucose and lipid uptake in BAT, although this effect was lost in the SD animals. In contrast, the glucose uptake in WAT in response to treatment with FGF21 was still evident in the SD state prior to body weight reaching a nadir. This suggests that the FGF21-induced increase in glucose uptake in WAT was independent of the whole-body changes in EE. This is in contrast to previous suggestions that the increase in thermogenic markers in WAT may stimulate glucose uptake via upregulation of GLUT4 [35].

We previously highlighted the importance of adiposity per se in governing FGF21 responsiveness and demonstrated that Siberian hamsters at the nadir of their body weight cycle following exposure to SD become completely unresponsive to exogenous FGF21 [16,17]. Here we extend those findings and demonstrate that at the nadir of the body weight cycle, KLB in all adipose tissue depots is reduced, and this is associated with a loss of ERK1/2 phosphorylation in response to treatment with FGF21. However, before body weight reaches a nadir, the animals remain FGF21 responsive. As SD exposure enhances sympathetic nervous system drive, increases UCP1 expression in BAT and WAT, and induces lipolysis, FGF21 may be unable to further drive this already maximal lipolytic and catabolic state at the nadir of the body weight cycle [42,43].

Our data do not exclude a role for FGF21 in the CNS. Previously, infusion of FGF21 into the lateral ventricle of mice was shown to stimulate the browning of AT, an effect that was attenuated in mice treated with a generic β-blocker and lost in mice lacking β-adrenoreceptors [14]. Further work is required to delineate the role of FGF21 in the brain of the current seasonal model of adiposity. A role for hypothalamic tanycytes in mediating the actions of FGF21 was recently proposed [44]. Indeed, we previously demonstrated a role for FGFR1c in tanycytes contributing to regulation of food intake, an effect probably mediated by tanycytes regulating the local availability of thyroid hormone in the surrounding hypothalamus [26]. In contrast to mice, which demonstrate increased food intake in response to treatment with FGF21, appetite in hamsters is consistently suppressed by FGF21 [3,16,17]. The FGF21-induced increase in appetite in mice is probably a compensatory response to enhanced whole-body energy expenditure, and therefore the defense of body weight or further metabolic fuel to drive thermogenesis. A suppressive action of FGF21 on appetite in hamsters is consistent with their known biology, in that a change in ingestive behavior is a major driver of their seasonal cycle of adiposity. Exposure of Siberian hamsters to SD for a period of weeks induces a profound (∼25%) chronic decrease in voluntary food intake, so hypophagia is a natural state in this species. In addition, this natural SD-induced hypophagia is associated with increased hepatic and brown fat production of FGF21 [45]. Correspondingly, peripheral treatment of Siberian hamsters with FGF21 results in upregulation of Dio2 in the hypothalamus; this enzyme converts thyroid hormone into its biologically active form [17]. Altered local concentration of thyroid hormone is considered the main hypothalamic driver of the seasonal cycle of appetite and energy expenditure in this species, and therefore it is a likely central pathway of action for FGF21 [46,47].

## 5 Conclusions

Taken together, our studies reveal novel insights into the primary sites and mechanisms of action of FGF21 using a preclinical natural model of adiposity, the Siberian hamster. They also highlight the importance of adipose tissue and KLB expression in governing the metabolic and homeostatic actions of FGF21, which produce differential effects in WAT and BAT, although we did not detect differences in the responses of subcutaneous and visceral WAT to FGF21. Our data indicate that glucose uptake in WAT may be used for de novo lipogenesis and TAG synthesis, whereas the selective increase in lipid uptake in BAT may fuel thermogenesis. These findings have important implications for FGF21's therapeutic potential in metabolic diseases that improve the understanding of this hormone and how it can be further exploited in future studies.

## Author contributions

JEL, HM, JL, and FJPE conducted the in vivo studies. AC and ACP developed the novel radioligands. JEL, CM, RG, and MF conducted the in vitro studies. JEL, CM, and SC conducted the gene/protein expression studies. JEL, FJPE, RJS, and KT designed the studies and wrote the manuscript. All authors revised the manuscript.

## Appendix A Supplementary data

**Supplemental Figure 1. FGF21 does not induce glucose and lipid uptake beyond adipose tissue.** (A) Glucose uptake of brain, liver, quadriceps muscle, and testes of the Siberian hamsters in LD treated with FGF21, vehicle, or pair-fed to match the food intake of the FGF21-treated group. Data are mean ± SEM. N = 4/group. Statistical analyses conducted using one-way ANOVA. (B) Lipid uptake of the brain, liver, quadriceps muscle, and testes of the LD Siberian hamsters treated with FGF21, vehicle, or pair-fed to match the food intake of the FGF21-treated group. Data are mean ± SEM. N = 4/group. Statistical analyses conducted using one-way ANOVA.

**Supplemental Figure 2. Siberian hamsters at the nadir of body weight after 12 weeks of exposure to short days (SD) are unresponsive to FGF21**. (A) Body weight loss, (B) food intake, (C) body weight, (D) RER, (E) energy expenditure, (F) ambulatory activity, (G-I) UCP1 and DIO2 mRNA expression, and (J) glucose uptake of the Siberian hamsters treated with FGF21, vehicle, or pair-fed to match the food intake of the FGF21-treated group. Data are mean ± SEM. N = 4/group.

**Supplemental Figure 3. Representative Western blotting underlying protein abundance depicted in Figure 2.** pERK1/2 activation (phosphorylation), total ERK, GLUT1, GLUT4 expression, ATGL, pHSL660, pACC, and control cyclophilin blots are depicted for interscapular brown fat (iBAT), subcutaneous white adipose tissue (sWAT), and visceral white adipose tissue (vWAT).
