# Supplementary figures and images for: Whole-body and adipose tissue-specific mechanisms underlying the metabolic effects of fibroblast growth factor 21 in the Siberian hamster
Source: Mol Metab. 2019 Nov 9;31:45–54. doi: 10.1016/j.molmet.2019.10.009 (PMC6889485; doi:10.1016/j.molmet.2019.10.009)

## Slide 1
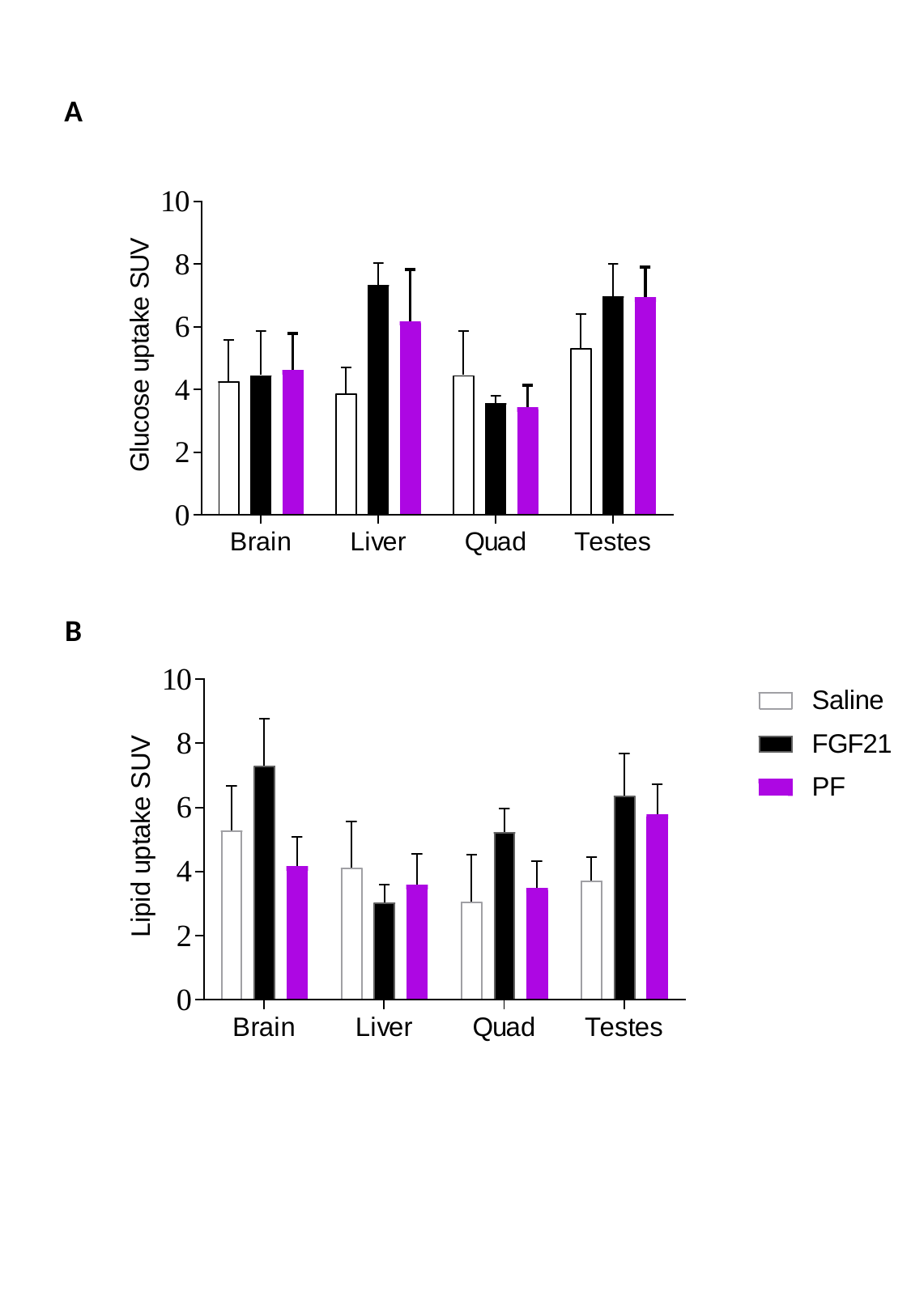

A
B

Supplement: Supplementary file 1 — Supplemental Figure 1. FGF21 does not induce glucose and lipid uptake beyond adipose tissue. (A) Glucose uptake of brain, liver, quadriceps muscle, and testes of the Siberian hamsters in LD treated with FGF21, vehicle, or pair-fed to match the food intake of the FGF21-treated group. Data are mean ± SEM. N = 4/group. Statistical analyses conducted using one-way ANOVA. (B) Lipid uptake of the brain, liver, quadriceps muscle, and testes of the LD Siberian hamsters treated with FGF21, vehicle, or pair-fed to match the food intake of the FGF21-treated group. Data are mean ± SEM. N = 4/group. Statistical analyses conducted using one-way ANOVA. [file mmc1.pptx]

## Slide 1
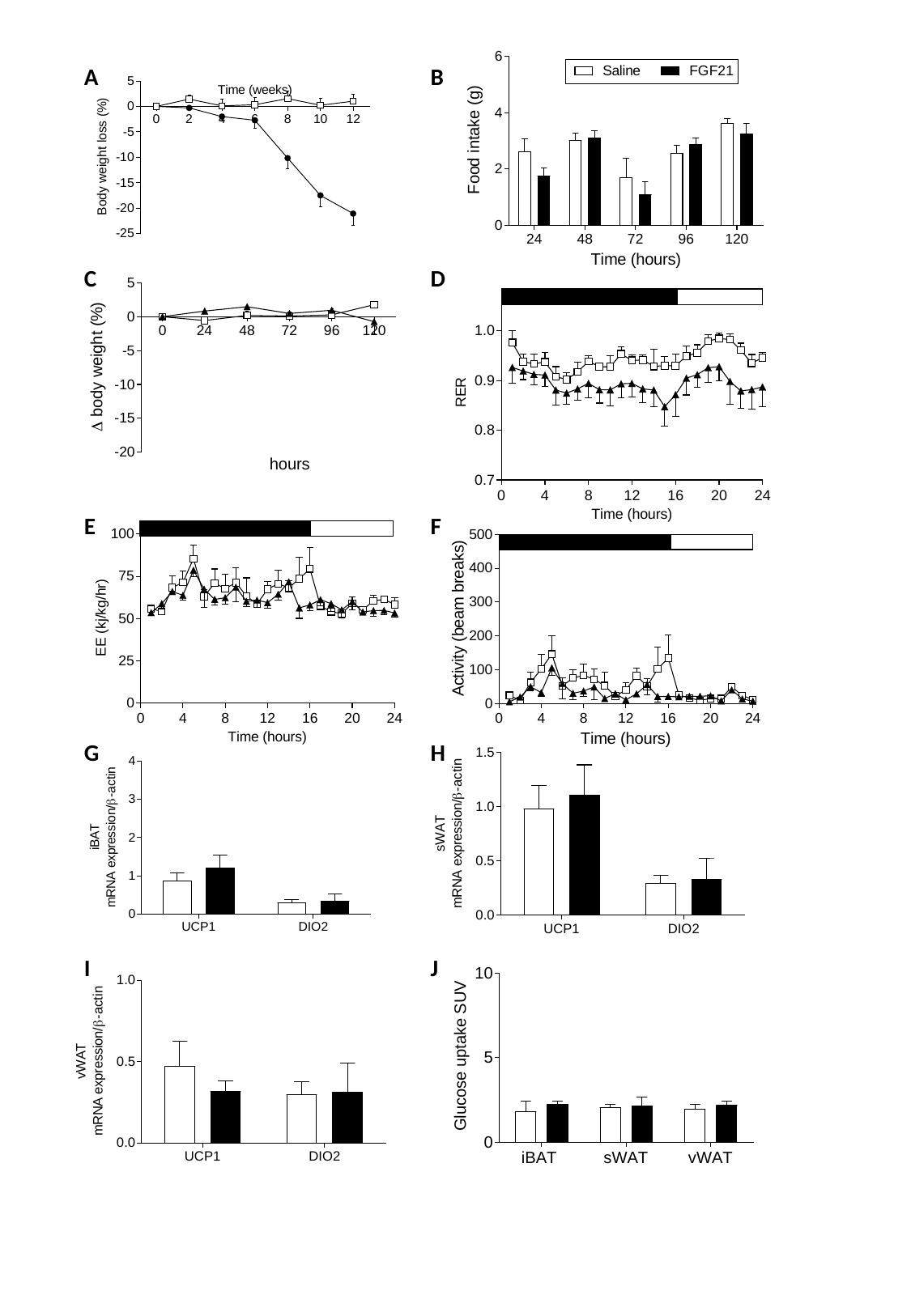

A
B
C
D
E
F
G
H
I
J

Supplement: Supplementary file 2 — Supplemental Figure 2. Siberian hamsters at the nadir of body weight after 12 weeks of exposure to short days (SD) are unresponsive to FGF21. (A) Body weight loss, (B) food intake, (C) body weight, (D) RER, (E) energy expenditure, (F) ambulatory activity, (G-I) UCP1 and DIO2 mRNA expression, and (J) glucose uptake of the Siberian hamsters treated with FGF21, vehicle, or pair-fed to match the food intake of the FGF21-treated group. Data are mean ± SEM. N = 4/group. [file mmc2.pptx]

## Slide 1
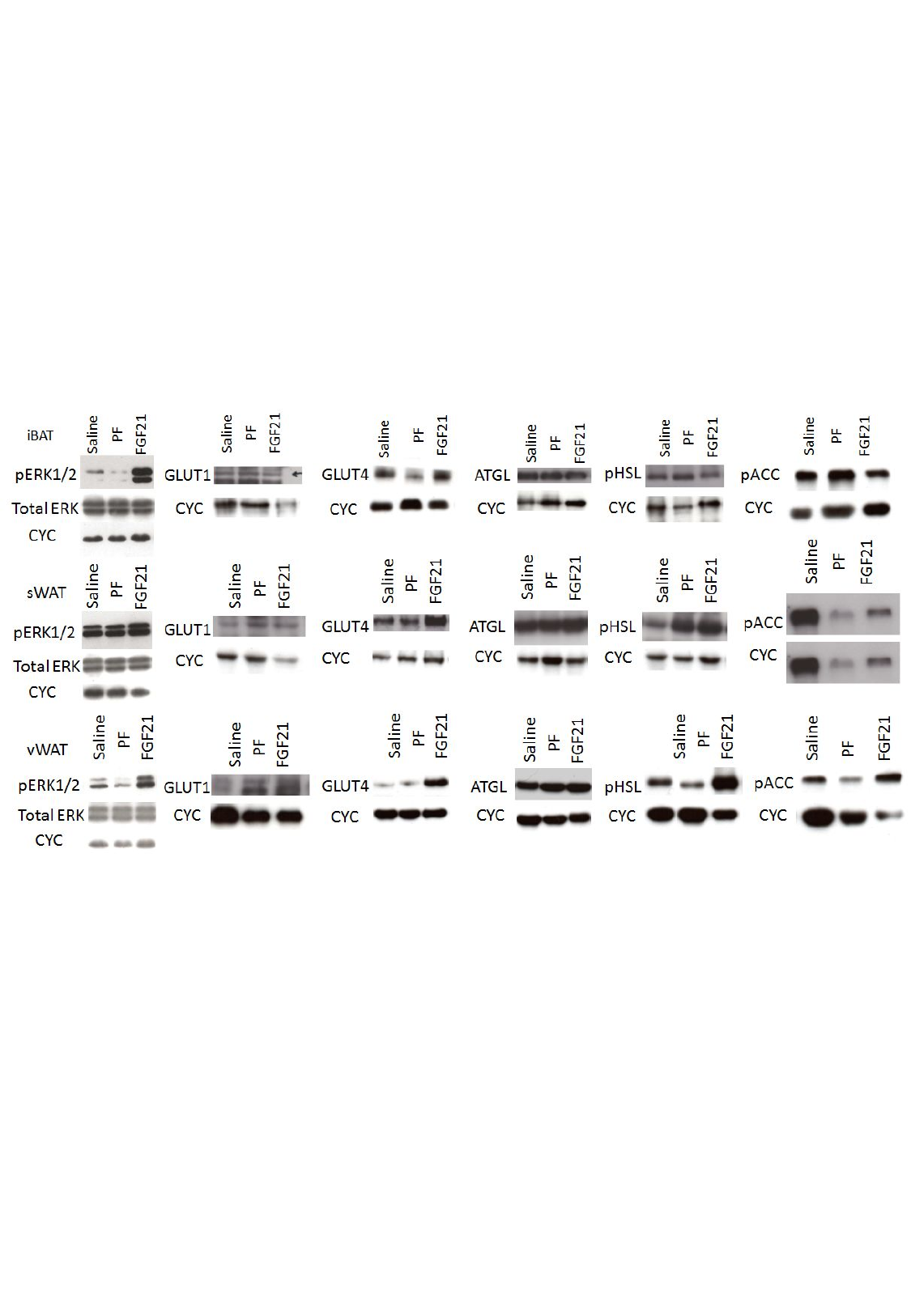

Supplement: Supplementary file 3 — Supplemental Figure 3. Representative Western blotting underlying protein abundance depicted in Figure 2. pERK1/2 activation (phosphorylation), total ERK, GLUT1, GLUT4 expression, ATGL, pHSL660, pACC, and control cyclophilin blots are depicted for interscapular brown fat (iBAT), subcutaneous white adipose tissue (sWAT), and visceral white adipose tissue (vWAT). [file mmc3.pptx]
