# Supplementary material for: Whole-body and adipose tissue-specific mechanisms underlying the metabolic effects of fibroblast growth factor 21 in the Siberian hamster
Source: Mol Metab. 2019 Nov 9;31:45–54. doi: 10.1016/j.molmet.2019.10.009 (PMC6889485; doi:10.1016/j.molmet.2019.10.009)
Supplement: Multimedia component 4 [file mmc4.docx]

**Supplemental Table 1. Antibodies.**

| **Peptide/protein target** | **Name of Antibody** | **Manufacturer, catalog #, and/or name of individual providing the antibody** | **Species raised in; monoclonal or polyclonal** | **Dilution used** | **RRID** |
| --- | --- | --- | --- | --- | --- |
| ATGL | Anti-Adipose Triglyceride Lipase antibody [EPR3444(2)] (ab109251) | Abcam, #ab109251 | Rabbit monoclonal | 1 in 1000 | AB_10864772 |
| phospho-ACC | Phospho-Acetyl-CoA Carboxylase (Ser79) Antibody #3661 | Cell Signalling, #3661 | Rabbit polyclonal | 1 in 1000 | AB_330337 |
| Cyclophilin B (PPIB) | Anti-Cyclophilin B antibody [k2E2] (ab74173) | Abcam, #ab74173 | Mouse monoclonal | 1 in 4000 | AB_1268387 |
| GLUT1 | Anti-Glucose Transporter GLUT1 antibody (ab652) | Abcam, #ab652 | Rabbit polyclonal | 1 in 2000 | AB_305540 |
| GLUT4 | Anti-Glucose Transporter GLUT4 antibody (ab33780) | Abcam, #ab33780 | Rabbit polyclonal | 1 in 2000 | AB_2191441 |
| phospho-HSL | Phospho-HSL (Ser660) Antibody #4126 | Cell Signalling, #4126 | Rabbit polyclonal | 1 in 1000 | AB_490997 |
| Actin | Anti-Actin antibody produced in rabbit (A2066) | Sigma, #A2066 | Rabbit polyclonal | 1 in 5000 | AB_476693 |
| phospho-ERK1/ERK2 | Phospho-p44/42 MAPK (Erk1/2) (Thr202/Tyr204) (D13.14.4E) XP® Rabbit mAb #4370 | Cell Signalling, #4370 | Rabbit monoclonal | 1 in 500 | AB_2315112 |
| Total ERK1/ERK2 | p44/42 MAPK (Erk1/2) (3A7) Mouse mAb #9107 | Cell Signalling, #9107 | Mouse monoclonal | 1 in 1000 | AB_10695739 |
| LPL | Anti-Lipoprotein lipase antibody [LPL.A4] | Abcam, #ab21356 | Mouse monoclonal | 1 in 1000 | AB_446221 |
| CD36 | CD36/SR-B3 Antibody | Novus, #NB400-144 | Rabbit polyclonal | 1 in 5000 | AB_10003498 |
| FATP1 | Anti-SLC27A1 antibody | Sigma, #SAB1402095-100UG | Rabbit polyclonal | 1 in 1000 | AB_10609989 |
| PPARα | Anti-PPAR alpha antibody | Abcam, #ab8934 | Rabbit polyclonal | 1 in 1000 | AB_306869 |
